# Markers of achievement for assessing and monitoring gender equity in translational research organisations: a rationale and study protocol

**DOI:** 10.1136/bmjopen-2015-009022

**Published:** 2016-01-07

**Authors:** Pavel V Ovseiko, Laurel D Edmunds, Linda H Pololi, Trisha Greenhalgh, Vasiliki Kiparoglou, Lorna R Henderson, Catherine Williamson, Jonathan Grant, Graham M Lord, Keith M Channon, Robert I Lechler, Alastair M Buchan

**Affiliations:** 1 Medical Sciences Division, University of Oxford, John Radcliffe Hospital, Oxford, UK; 2 National Initiative on Gender, Culture and Leadership in Medicine: C-Change, Brandeis University Women's Studies Research Center, Waltham, Massachusetts, USA; 3 Nuffield Department of Primary Care Health Sciences, University of Oxford, Oxford, UK; 4 NIHR Oxford Biomedical Research Centre, Joint Research Office, Churchill Hospital, Oxford, UK; 5 Women's Health Academic Centre, King's College London, Guy's Hospital, London, UK; 6 NIHR Biomedical Research Centre at Guy's and St Thomas’ NHS Foundation Trust and King's College London, Guy's Hospital, London, UK; 7 Guy's and St Thomas’ NHS Foundation Trust, Guy's Hospital, London, UK; 8 Policy Institute, King's College London, London, UK; 9 MRC Centre for Transplantation, King's College London, Guys’ Hospital, London, UK; 10 Oxford University Hospitals NHS Foundation Trust, John Radcliffe Hospital, Oxford, UK; 11 King's Health Partners, Guy's Hospital, London, UK

**Keywords:** Gender equity, Translational research, NIHR Biomedical Research Centre (BRC), Evaluation, Research impact assessment

## Abstract

**Introduction:**

Translational research organisations (TROs) are a core component of the UK's expanding research base. Equity of career opportunity is key to ensuring a diverse and internationally competitive workforce. The UK now requires TROs to demonstrate how they are supporting gender equity. Yet, the evidence base for documenting such efforts is sparse. This study is designed to inform the acceleration of women's advancement and leadership in two of the UK's leading TROs—the National Institute for Health Research (NIHR) Biomedical Research Centres (BRCs) in Oxford and London—through the development, application and dissemination of a conceptual framework and measurement tool.

**Methods and analysis:**

A cross-sectional retrospective evaluation. A conceptual framework with markers of achievement and corresponding candidate metrics has been specifically designed for this study based on an adapted balanced scorecard approach. It will be refined with an online stakeholder consultation and semistructured interviews to test the face validity and explore practices and mechanisms that influence gender equity in the given settings. Data will be collected via the relevant administrative databases. A comparison of two funding periods (2007–2012 and 2012–2017) will be carried out.

**Ethics and dissemination:**

The University of Oxford Clinical Trials and Research Governance Team and the Research and Development Governance Team of Guy's and St Thomas’ National Health Service (NHS) Foundation Trust reviewed the study and deemed it exempt from full ethics review. The results of the study will be used to inform prospective planning and monitoring within the participating NIHR BRCs with a view to accelerating women's advancement and leadership. Both the results of the study and its methodology will be further disseminated to academics and practitioners through the networks of collaborating TROs, relevant conferences and articles in peer-reviewed journals.

**Strengths and limitations of this study**

- The study addresses the previously neglected need to assess and monitor gender equity in translational research organisations with a view to accelerating women's advancement and leadership.
- We anticipate that an adapted balanced scorecard approach will enable clarification and translation into operational terms of the organisation's vision and strategy regarding gender equity.
- The reliability and validity of this cross-sectional retrospective study will depend on the completeness and accuracy of historical data sets and the practicalities of data extraction from these.
- If this approach proves feasible and robust in a two-centre study, other centres will be encouraged to apply the same methodology to generate comparative data.

## Introduction

Translational research organisations (TROs) are important elements of the UK's expanding research infrastructure, with a remit to translate biomedical discoveries into effective therapies for patients. Whereas other industries have long recognised that ‘winning the talent war for women’ is key to their growth,^1^ the lack of gender equity in academic medicine remains a serious threat to the quality and international competitiveness of translational research. Underutilisation of women's talent and potential in biomedical research, especially at senior levels and in leadership roles, has been well documented.^2–4^ This deprives biomedical research of women's perspectives^5^ ^6^ and more collaborative leadership styles.^7^ Moreover, some fields (notably women's and paediatric health) are less likely to be investigated by men than women.^8^ A relative dearth of women mentors and role models in senior positions not only slows down the advancement of the current generation of women translational researchers, but also presents a major problem for the education and training of the next generation. Finally, gender inequity may be the manifestation of discriminatory practices and unconscious biases,^2^ for which there should be no place in today's science and society. Participation of both genders fairly and without bias is imperative for the legitimisation of and public support for science, including allocation of public resources.^9^ For all these reasons, TROs need to demonstrably accelerate women's advancement and leadership.

In England, the National Institute for Health Research (NIHR) has challenged NIHR-funded TROs to improve gender equity and career advancement for women in biomedical research.^10^ For example, NIHR Biomedical Research Centres and Units (BRCs and BRUs) are not expected to be short listed and therefore eligible for future funding ‘where the academic partner (generally the Medical School/Faculty of Medicine) has not achieved at least the Silver Award of the Athena SWAN Charter for Women in Science’.^11^ The Charter's awards recognise different levels of commitment to advancing women’s careers in science, technology, engineering, maths and medicine (STEMM) based on 10 principles.^12^

While Athena SWAN awards are useful markers of achievement for higher education institutions and research institutes, they alone are likely to be insufficient for assessing and monitoring the progress of NIHR TROs towards gender equity—not least because they were not designed for such a purpose. We believe that NIHR TROs need to make their own measurable contribution to accelerating women's advancement and leadership in translational research. Our study addresses this previously neglected need by focusing specifically on two of the UK's leading TROs—the NIHR BRC at the Oxford University Hospitals National Health Service (NHS) Foundation Trust and the University of Oxford and the NIHR BRC at Guy’s and St Thomas’ NHS Foundation Trust and King’s College London. This approach is likely to be relevant and transferable to other TROs in the UK and (with adaptation) comparable organisations around the world.

## Methods and analysis

### Study overview

This study has two components. The first is the development of a conceptual framework to assess and monitor gender equity in the participating NIHR BRCs. This paper describes the protocol for the development of the conceptual framework, which will be refined with an online stakeholder consultation and semistructured interviews to test the face validity and explore practices and mechanisms that influence gender equity in the given settings. The second component is the application of the conceptual framework in repeated cross-sectional evaluations resulting in a historical picture of how things have changed over time. Devising and applying a framework will ensure that this process is done consistently on each occasion and provide comparable observational data. It will also provide an indication of the quality and consistency of data over the study time period and the feasibility and resource requirements for obtaining and analysing these data.

### Study aim and objectives

The aim of the study is to inform the acceleration of women's advancement and leadership in translational research through the development, application and dissemination of a conceptual framework and measurement tool. The objectives of the study are as follows:

- To devise a conceptual framework which captures the major dimensions of gender equity in the NIHR BRCs and complements the existing forms of performance monitoring and research impact assessment.
- To assess data using this framework from the administrative databases for the two previous funding rounds (2007–2012 and 2012–2017) within the NIHR BRCs.
- To create an objective baseline and evidence base that informs prospective planning and monitoring of gender equity.

### Study setting

The study will be conducted at two NIHR BRCs—partnerships between the UK's leading NHS organisations and universities. Together with the other NIHR-funded TROs, such as BRUs and Collaborations for Leadership in Applied Health Research and Care (CLAHRCs), NIHR BRCs are part of the UK Government's initiative to enable a more effective translation of basic science discoveries to clinically testable interventions, especially drugs and devices.^13^

The NIHR BRC at the Oxford University Hospitals NHS Foundation Trust and the University of Oxford was established in 2007 through a competitive NIHR award of £57 million over 5 years, and in 2012 it was awarded a further 5 years funding of £96 million. This funding enables NHS consultants and university academics to devote time and resources to concentrating on translational research across a number of themes. Currently, there are nine ‘vertical’ research themes that have a disease or therapeutic focus (eg, cardiovascular, diabetes, vaccines); five ‘cross-cutting’ research themes that bring together platform technologies (eg, genomic medicine, biomedical informatics and technology, surgical innovation and evaluation); and seven working groups that are set up to develop strategic priorities (eg, cognitive health, molecular diagnostic, and research education and training; http://oxfordbrc.nihr.ac.uk).

The NIHR BRC at Guy’s and St Thomas’ NHS Foundation Trust and King’s College London was also established in 2007, being awarded £51 million. It successfully bid for a further £68 million in 2012 to fund its work until 2017. This NIHR BRC focuses on taking advances in basic science out of the laboratory and into clinical settings in order to benefit patients at the earliest opportunity, and creates an active partnership between clinical and academic staff. The eight research themes cross-cut with four clusters, which focus on different stages of translational science (http://www.guysandstthomasbrc.nihr.ac.uk).

### Study population

Unlike Athena SWAN, which focuses on university academic and research staff, our study population is intentionally broader and includes all NHS consultants and university clinical academics funded by the NIHR BRCs (ie, NIHR investigators and NIHR senior investigators); administrative and support staff (ie, NIHR associates); academic and clinical trainees (ie, NIHR trainees); and leaders (including the most senior executive and non-executive committees in both NIHR BRCs). These professional groups have been selected because of their involvement in translational research as well as its administration, support and leadership. Their numbers are also sufficiently high to warrant a retrospective evaluation and prospective planning.

### Conceptual framework development

Although a comprehensive literature search returned no directly relevant instruments for TROs, we identified the US National Initiative on Gender, Culture and Leadership in Medicine (C-Change) at Brandeis University (http://cchange.brandeis.edu) as an example of current best practice, and established collaboration with the authors of the C-Change Markers of Achievement Index (MAI). This instrument was designed and used in five US medical schools to track temporal patterns indicating progress in leadership and achievement for female faculty and of faculty from racial and ethnic groups under-represented in US medicine.^14^ One of the authors (LHP), who heads C-Change, shared the C-Change MAI instrument used in US medical schools during the 2014 Mid-Term Review of the NIHR Oxford BRC.

**Figure 1:**
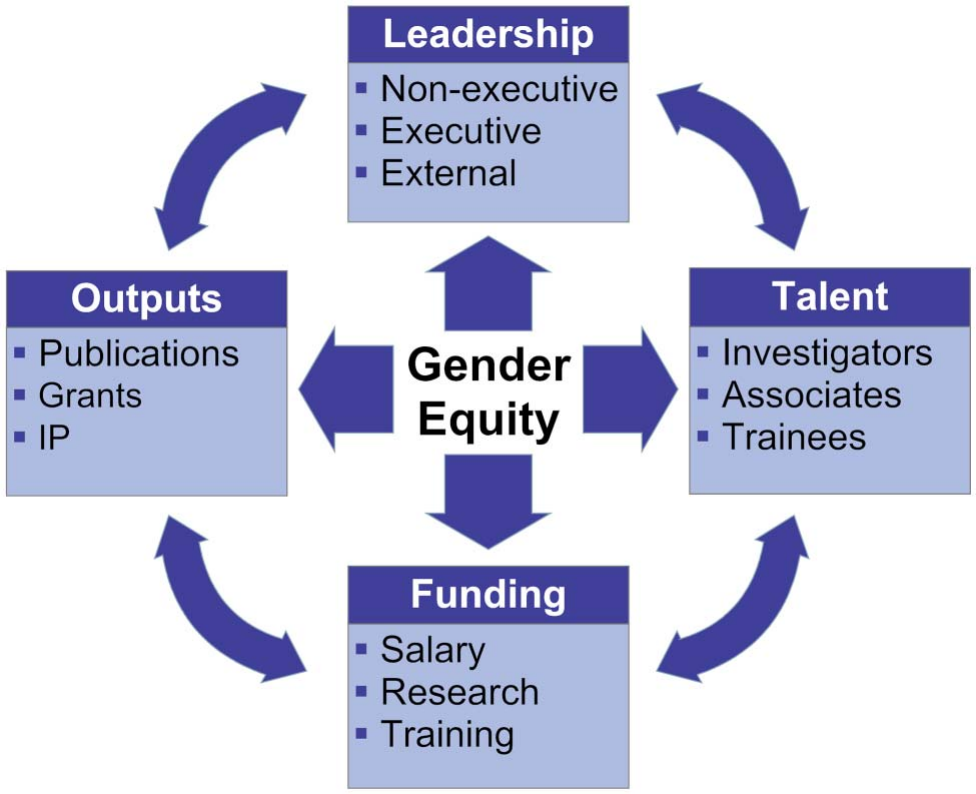
Multidimensional conceptual framework for gender equity assessment and monitoring. IP, intellectual property.

We developed our conceptual framework (figure 1) by adapting the C-Change MAI instrument and a wider relevant literature using the balanced scorecard approach proposed by Kaplan and Norton.^15^ The advantage of the balanced scorecard, widely used in the commercial sector and in US healthcare organisations, is its departure from the traditional performance assessment based solely on financial measures, to include intangible assets and capabilities needed for future growth.^15^ The ‘scorecard’ comprises two backward-looking measures of what has already been achieved (in the original model, value for customers and financial profit) and two forward-looking measures of process (effective and efficient work practices, and learning and growth). This approach enables organisations to clarify their vision and strategy and then translate these into operational terms.^16^ It has previously been adapted for performance assessment in TROs by Pozen and Kline,^17^ in academic clinical departments by Rimar and Garstka,^18^ and in a national health system by El Turabi *et al*.^19^ We draw on their work in adapting the balanced scorecard to look at gender equity as an important area of performance improvement and research impact assessment in TROs.

In keeping with the balanced scorecard approach,^15–17^ our conceptual framework distinguishes between several levels of performance assessment, planning and monitoring:

- *Performance dimensions* represent which domains TROs need to address to promote gender equity in a balanced way;
- *Markers of achievement* are specific measures of these performance dimensions;
- *Metrics* translate markers of achievement into operational terms and highlight what is important to a given TRO;
- *Targets and milestones* express the TRO's goals and strategic objectives, and help monitor progress in achieving them.

The effectiveness of the conceptual framework will be determined according to the SMART criteria (Specific, Measurable, Assignable, Realistic, Time-related)^20^ and in compliance with the UK Government's FABRIC framework for performance information systems (Focused, Appropriate, Balanced, Robust, Integrated, Cost-Effective).^21^ Below we explain the first three levels of the conceptual framework (table 1). Targets and milestones pertaining to the fourth level of the conceptual framework will be established as part of the actual planning process during the next funding period, which is beyond the scope of the current study.

**Table 1:** Markers of achievement and candidate metrics for gender equity assessment and monitoring

Performance dimensions	Markers of achievement	Candidate metrics
Leadership	Non-executive leadership roles Executive leadership roles External leadership roles and esteem indicators	Absolute and relative numbers of women with non-executive and executive roles Absolute and relative number of women with external leadership roles and esteem indicators such as membership in professional and scientific organisations, panels, and societies Absolute and relative numbers of women promoted to executive leadership roles from within the organisation Availability and effectiveness of policies and programmes aimed at gender-sensitive leadership development, retention and succession planning
Talent	Investigators Associates Trainees	Absolute and relative numbers of women investigators and senior investigators, associates, and trainees Absolute and relative numbers of women investigators and senior investigators who are substantively employed as NHS consultants and university academics Organisational efforts aimed at the recruitment and retention of women Opportunities for women to advance their careers within the organisation
Funding	Salary costs Research costs Training	Absolute and relative numbers of women in receipt of BRC funding towards investigators’ salary (programmed activities), research costs and training Average and total amounts of different types of BRC funding received by gender BRC funding application success rates by gender
Outputs	Publications Grants and contracts Intellectual property	Absolute and relative numbers of publications by women according to the type of contribution (first/senior author and any contribution) and the type of publication (high-impact factor journal and any journal) Number of times publications by women and men have been cited Absolute and relative numbers of patents applied for and granted, number of spin-out companies established, and overall income from intellectual property Absolute and relative numbers of grants and contracts and amount of funding awarded to women

BRC, Biomedical Research Centre; NHS, National Health Service.

#### Leadership

Leadership is key to a TRO's ability to identify research questions and develop research projects that are of great importance to society and can have high translational impact. There are many styles of leadership and diversity of perspectives, which more women in senior positions can make available.^6^ ^22^ For example, studies show that women are more likely than men to exhibit transformative leadership styles, which employ more collaborative and less hierarchical approaches.^7^ Although differences between male and female leaders may be minimal, the characteristics of leadership style on which women exceeded men are positively associated with leaders’ effectiveness,^7^ with higher performance and better outcomes in healthcare,^23–25^ and with the collaborative and team culture preferred by physician scientists in a given setting.^26^ ^27^ Additionally, leaders serve as mentors and role models for the next generation of leaders—so more female leaders in TROs today may mean more transformative leadership styles used by both male and female leaders of tomorrow.^28^ Even though studies show gender equity at medical school entry and equal leadership aspirations in academic medicine among men and women faculty, the under-representation of the latter in academic medicine leadership roles persists.^2^ ^29–31^

Markers of achievement for leadership concern women leaders in both non-executive and executive roles. Candidate quantitative metrics include both the absolute and relative numbers of women leaders in post, women promoted to leadership roles from within the organisation, and women leaders recruited from the outside of the organisation. Other candidate metrics include the availability and effectiveness of policies and programmes aimed at gender-sensitive leadership development, retention and succession planning. Candidate metrics may also include external leadership roles and esteem indicators such as membership of professional and scientific organisations, panels and societies.

#### Talent

Talent, by which we mean individuals’ experience and capability in basic and/or translational science, is the cornerstone of a TRO's ability to translate biomedical discoveries into benefits for patients in a creative, cost-effective and timely manner. Studies show that many women are attracted to academic medicine because of their interest in clinical work with patients.^32^ Therefore, an increase in the number of women researchers is likely to have a positive influence on the clinical focus of biomedical research. Likewise, women in academic medicine appear to be interested in teaching^33–35^ and mentoring^36^ more than men, though these differences may not be due to innate traits. An increase in the number of women is therefore likely to enhance the ability of a given TRO to train and mentor the next generation of translational researchers. However, clinical work and teaching have a lower status than research, so engaging with them can impede women's advancement and leadership in TROs. Studies also show that female faculty in academic medicine have less alignment of their personal values with undesirable behaviours often observed in academic medicine, for example, devaluing of social and clinical missions of academic medicine, questionable ethical behaviour, and the necessity for self-promotion to achieve success.^31^ ^37^ ^38^ This may suggest that having more women faculty would result in a more values-based approach to research. Research beyond medicine shows the importance of having a critical mass of women as a predictor of their acceptance and success.^9^ In organisations (outside medicine), where women make up fewer than 15% of the workforce, women are less likely to be accepted in the organisation and less likely to progress their careers.^39^

Markers of achievement for talent concern women in several different categories, including investigators and senior investigators who are directly involved in undertaking research; associates who support and administer research led by others; and trainees who undertake research training and career development.^40^ Candidate quantitative metrics for faculty members include both the absolute and relative numbers of women in different categories. Another important candidate metric is the proportion of women investigators who are substantively employed as NHS consultants and university academics. Candidate qualitative metrics include organisational efforts aimed at the recruitment and retention of women as well as opportunities for women to advance their careers within the organisation.

#### Funding

Funding supports a given TRO to sustain all aspects of its activities, ranging from resourcing research projects, buying out time of NHS consultants to conduct research, providing research training and administering research. Research from both US and European academic medicine suggests that women are awarded fewer grants and of lesser value compared with men.^41–45^ This lack of parity in funding may indicate not only overt gender differences in the levels of productivity and in certain research areas, but also more covert aspects at play such as gender discrimination^46–49^ and unconscious bias.^50^ ^51^ Therefore, greater parity in the allocation of funding between men and women will give women more equitable opportunities to conduct research as well as indicate progress towards freedom from gender discrimination and bias.

Markers of achievement for funding concern women in receipt of different streams of BRC funding, including the main stream of funding that goes towards individual research projects, but also a stream of funding towards buying out NHS consultants’ time (programmed activities) to conduct research, and a stream of funding towards research training awards for academic fellows and research nurses. Candidate quantitative metrics for funding include both the absolute and relative numbers of women in receipt of different streams of BRC funding, average and total amounts of funding received by gender, as well as funding application success rates.

#### Outputs

Outputs express the research-based contribution of a given TRO to the health of local and global patient communities and to the nation's economic and social development. Although there is substantial evidence to suggest that women publish less than men,^52–57^ there is also evidence to suggest that women publish as much as men,^58–64^ particularly after adjusting for age and rank.^49^ ^65^ Moreover, women's publications rates are increasing^57^ ^66^ ^67^ and can actually exceed men's publication rates in the latter stages of careers.^62^ Therefore, an increase in the number of research outputs by women will indicate an improved institutional capacity to utilise women's talent in translational research and to support women at different stages of their careers.

Markers of achievement for research outputs concern all major types of research outputs by women faculty, including not only publications, but also intellectual property and external grants and contracts. Candidate quantitative metrics for publications include both absolute and relative numbers of publications by women according to the type of contribution (first/senior author and any contribution) and the type of publication (high-impact factor journal and any journal) as well as the number of times publications by women and men have been cited. Candidate metrics for intellectual property include both absolute and relative numbers of patents applied for and granted, number of spin-out companies established, and overall income from intellectual property. Candidate metrics for external grants and contracts include the absolute and relative numbers of grants and amount of funding awarded to women.

### Face validity

In order to ensure that all the relevant elements of the proposed conceptual framework and markers of achievement are identified and included in the study, their face validity will be established. The latter denotes the degree to which the contents of the test and its items, which in our case is the conceptual framework with markers of achievement and metrics, are viewed by test respondents as relevant to the context in which the test is being conducted.^68^ Therefore, we will consult a panel of stakeholders representing the entire study population and then adjust the conceptual framework and its elements according to their feedback.

The consultation will be carried out using an anonymous online survey and face-to-face semistructured interviews. The survey instrument will comprise both closed-ended quantitative and open-ended qualitative questions. Respondents will be asked to critically appraise the proposed conceptual framework with markers of achievement and metrics (table 1) as well as elaborate on practices and mechanisms that influence gender equity in the given settings. The survey instrument will also include demographic information, including leadership and staff group, substantive employment, gender, and age. Interviewees will be purposively selected to achieve a representative sample of the study population. An adequate interview sample size will be based on the number of interviews necessary to achieve a saturation point, that is, when additional interviews provide no new themes or categories.

### Data collection

Data will be collected from the relevant administrative databases within the participating NIHR BRCs in two phases. Phase I for the funding period 2007–2012 will start in Q1 2016 with a consultation with a panel of NIHR investigators, NIHR associates and leaders to establish the face validity of the proposed conceptual framework with markers of achievement and metrics. Phase II for the current funding period (2012–2017) will start in Q2 2016 to coincide with the beginning of planning for the next BRC 5-year funding application (2017–2022).

### Data analysis

In order to carry out a retrospective evaluation of the two funding periods, yearly data collected from the relevant databases will be aggregated for each of the two 5-year funding periods and analysed statistically within Excel and SPSS. Individual variables will be based on the metrics identified in the framework (figure 1). Descriptive statistics will be used to present data pertaining to markers of achievements in each of the funding periods. Tests of significance will be used to carry out comparisons between the current and previous funding periods and to detect gender differences within a given funding period. Missing data are a common occurrence with observational data; however, at present the nature of any missing data is unknown, that is, whether it is random or non-random. Once this is established, the appropriate method to address this will be applied.^69^

In order to aid priority setting and quantification of markers of achievement during the next funding period, comparisons between the two funding periods will be used. To aid planning further, comparisons between the participating NIHR BRCs and their founding academic and clinical partners will be made. Such comparisons are important because women's advancement and leadership within both NIHR BRCs are enabled and constrained by the pool of qualified women within the NHS Trusts and the Universities. For example, the extent to which the number of women investigators within each NIHR BRC can be increased will depend on the number of qualified women consultants and clinical academics in the NHS Trust and the University, respectively.

## Ethics and dissemination

The University of Oxford Clinical Trials and Research Governance Team and the Research and Development Governance Team of the Guy’s and St Thomas’ NHS Foundation Trust reviewed the study and deemed it exempt from full ethics review on the grounds that it falls outside of the Governance Arrangements for Research Ethics Committees (GAfREC), which stipulate which research studies are required to have ethics review. Once the survey instrument is finalised, it will be assessed against the standards set out in the Central University Research Ethics Committee (CUREC) Checklist 1, and if necessary submitted to the CUREC for review. All data collected from the relevant administrative databases will be held and analysed in compliance with the requirements of the UK Data Protection Act 1998 and other relevant legislation and professional guidance. During analyses, data will be aggregated and anonymised. The results of the study will be published internally within both BRCs and used to inform planning with a view to accelerating women's advancement and leadership. Both the results of the study and its methodology will be further disseminated to academics and practitioners through the networks of collaborating TROs, relevant academic and clinical forums, conferences, and articles in peer-reviewed journals.

## Discussion

### Rationale

The fundamental rationale for this study stems from the desire of the participating NIHR BRCs to make their own measurable contribution to accelerating women's advancement and leadership in translational research. We extend previous work on performance assessment in translational research,^17–19^ ^70–72^ by focusing on gender equity. Our intention is to develop a new multidimensional conceptual framework for gender equity performance assessment in order to use it both for retrospective evaluation and prospective planning and monitoring with a view to accelerating women's advancement and leadership. We believe that measuring the impact of TROs on gender equity should become an important dimension of their research impact assessment.

### Strengths and limitations

This study has several strengths and limitations. First, it addresses the previously neglected need to assess and monitor gender equity in TROs, focusing specifically on two of the UK's leading TROs. Although there is the need to accelerate women's advancement and leadership in TROs, empirical studies addressing this need remain scarce, especially in the UK. To the best of our knowledge, this is the first study to empirically investigate gender equity in NIHR BRCs in the UK.

Second, an adapted balanced scorecard approach enables clarification and translation into action of the organisation's vision and strategy regarding gender equity. This will enable the participating NIHR BRCs to identify areas where improvements are needed and inform planning during the next funding period. In doing so, the balanced scorecard approach will help NIHR BRC leaders to operationalise the organisation's ambitions for accelerating women's advancement and leadership as well as establishing targets and milestones for monitoring progress against the goals and strategic objectives.

Third, the inclusion in the online stakeholder consultation and semistructured interviews of the questions regarding practices and mechanisms that influence gender equity in the given settings will help identify potential strategies to accelerate women's advancement and leadership. The current evidence base for such strategies is predominantly based on observational studies from North America. Our study will help identify interventions that are most relevant to UK TROs and will propose a rigorous tool to measure their efficacy as part of prospective organisational development and change.

Fourth, the reliability and validity of this cross-sectional retrospective study will depend on the completeness and accuracy of historical data sets and the practicalities of data extraction from these. The study will rely on data extraction from data sets across two different types of institutions, a university and a healthcare provider organisation. Their information systems use different definitions and data codes, and were not specifically designed for assessing and monitoring gender equity. These may limit the completeness and accuracy of the aggregated data.

Finally, if this approach proves feasible and robust in a two-centre study, other centres will be encouraged to apply the same methodology to generate comparative data. NIHR BRUs and NIHR CLAHRCs in the UK as well as similar organisations around the world could use the results of the study in order to benchmark their own organisations against the two NIHR BRCs. This may facilitate organisational learning between different TROs and lead to further research seeking to determine comparatively the most effective strategies to accelerate women's advancement and leadership.

### Implications and conclusions

This study defines and tests a new tool for assessing gender equity in two of the UK's leading NIHR BRCs. The results of the study will inform strategic planning and monitoring during the next funding period with a view to accelerating women's advancement and leadership in the participating NIHR BRCs. In doing so, the study will develop new processes and information systems for the collection and analysis of data on gender equity. These processes and information systems will be refined further through continuous feedback from strategic planning and decision-making. The study will also have wider implications. If the methodology, processes and information systems developed as part of the study prove effective, they can be applied to the other neglected dimensions of equity in translational research such as race and ethnicity, disability, sexual orientation, and age.
